# Experiments and Observations on Hanging

**Published:** 1838-04

**Authors:** 

**Affiliations:** Berlin


### PART FIFTH.

### Medical Intelligence.

---

### EXPERIMENTS AND OBSERVATIONS ON HANGING. BY DR. CASPER, OF BERLIN.

THE principal object of these experiments was to determine whether any evidence of hanging during life could be obtained from the *impression left by the cord* on the neck. Dr. Casper has derived his observations from medico-legal practice, as well as from experiments on rabbits and recently dead human subjects. The results at which he has arrived correspond with those of Orfila and Beaudè, previously published in France. They completely refute the ancient notion, relative to the constant production of ecchymosis by the cord in the *living;* an error which, however, is still adopted and acted on by numerous modern medical practitioners. The other and even more serious mistake of the non-production of ecchymosis, being regarded as a certain proof of hanging after death, is also strikingly exposed.

The following remarkable case was communicated to the author by Dr. Hinze, of Waldenburg, in the year 1826:

A young man, in a fit of drunkenness, hung himself with a stout cord. He was cut down in about half an hour afterwards; and Dr. Hinze and others endeavoured to resuscitate him. The cord had merely produced a superficial mark on the neck, destitute of any appearance of ecchymosis. Signs of returning animation began to manifest themselves, the efforts of the medical attendants were continued for several hours, but the traces of vital reaction disappeared. To the astonishment of all present, when life was about to become again extinct, the *mark* on the neck became *deeply ecchymosed.* This ecchymosed condition was verified by an inspection made the following day. Death was due to sanguineous apoplexy.

Dr. Casper commenced by performing some experiments on rabbits; but we hold all such experiments to be utterly valueless, in a practical point of view. The skin of animals differs too widely from that of a human being to allow of any safe analogical inferences being drawn from observations of this kind. At any rate, we think a court of law would have fair ground for objecting to the opinions of that witness whose evidence was influenced by such experiments. Several instances are quoted, in which hanging a body has been resorted to by a murderer for the purpose of concealing his crime. The two following cases Dr. Casper met with in his own practice.

A boy was found hanging perfectly dead. On examination, a round ecchymosed mark, about the size of a dollar, was seen on the larynx, with several impressions on the surrounding skin. There was neither depression nor ecchymosis in the course of the cord around the neck. The inspection left no doubt that the deceased had died from asphyxia; and it was discovered that the boy had been first strangled and afterwards hanged.

In the second case, a man, sixty years old, was found hanging in a room. His body was so suspended to a hook in the door, that the nates were not more than nine inches from the floor; and his legs were stretched out at full length. The cord by which he was suspended was from two to three feet long, and was loosely passed around his neck. The furniture of the room was in great disorder, and some marks of dried blood were seen on one part of the floor. The right side of the head and face of the deceased was in several parts ecchymosed and excoriated. A circular impression had been produced in the neck by the cord; but there was no extravasation beneath. A little above this was a strongly ecchymosed mark, extending round about one-half of the neck to the occiput. In the skin beneath this, blood was found extravasated. Death was owing to asphyxia. The medical opinion expressed, was to the effect that the deceased had been first murdered and afterwards hanged. Evidence subsequently adduced confirmed this opinion.

Dr. Casper's experiments on the dead human subject amounted to eight.

1. A man, æt. twenty, *an hour* after death from typhus, was hanged by a double cord passed above the larynx. In about twenty-four hours the body was cut down and examined. Around the neck between the larynx and os hyoides was a double parallel mark about three lines deep, of a *brown colour with a slight tinge of blue.* There were traces of cadaverous ecchymosis about the body. The whole appearance of this subject was such that any individual, not acquainted with the circumstances, would have supposed on looking at it that the deceased had been hanged while living. Some spots on the right side of the neck were strongly coloured. The skin of this part was hard, like leather; and in patches slightly excoriated. There was no extravasation of blood in the cellular texture; but the muscles of the neck beneath were of a deep violet colour. The large vessels of the neck were not congested.

2. A young man, æt. twenty-three, *an hour* after death from phthisis was hanged with a double cord, and his body was examined on the following day. A double depression was seen around the neck, in which were marks produced by the prominent portions of the cord. The depressions were of a *yellowish brown colour without ecchymosis.* The cutis was as if burnt and like parchment, both when felt and cut. There was no blood extravasated in the cellular tissue beneath.

3. A man, æt. seventy, who died from dropsy was hanged *two hours* after death. The impressions on the neck presented the same characters as in the preceding case.

4. The next subject was a man who died of apoplexy. *Thirteen hours* after death, a cord was very tightly drawn around the neck above the larynx. Six hours afterwards, on examining the constricted part, a soft impression, easily removed by pressure, was perceptible. There was no colour, nor was there any change whatever in the skin.

5. *Six hours* after death the double cord was tightly drawn around the neck of a female above the larynx. On the following morning it was removed, and the skin examined. There was no particular appearance, the part where the cord had been applied was scarcely distinguishable.

6. *Twenty-four hours* after death, the double cord was very tightly drawn around the neck of a man. On the following day, a slight double depression was perceptible: but there was no alteration of colour, nor any change in the skin either on the surface or beneath.

7. In a similar experiment, after the same period of time, the spot where the cord had been applied could scarcely be seen.

8. The last experiment was on the body of a child about a year and a half old. On the day after death, a small cord was tightly drawn and secured around the neck. Twenty-four hours afterwards, it was found that a small bluish-coloured mark had been produced by the constriction. This mark, although very superficial, was still visible enough to strike the eye. There was no trace of extravasated blood on cutting into it.

Before giving a summary of conclusions from his experiments, the author presents us with an interesting statistical account of 106 cases of hanging, the greater number of which are borrowed by him from various medico-legal reports. Out of the 106 cases, there were 77 males and 29 females. The means of suspension used were various:

| | |
|---|---|
| A cord was employed in . . . . | 51 |
| Handkerchiefs, bandages, stockings, in . | 25 |
| Unknown means . . . . . | 30 |

In sixteen cases out of nineteen, it was found that the use of a handkerchief did not prevent the formation of a visible mark around the neck; and on the whole, the author thinks that the article used for suspension has but little influence on the local changes produced. The exact site of the ligature is also of little consequence, either in respect to the formation of a mark by the cord, or to the manner in which death takes place, i. e. whether by apoplexy or by asphyxia. In regard to the position of the ligature it was found to be:

| | |
|---|---|
| Between the os hyoides and larynx in . . | 59 |
| On the larynx or thyroid cartilage in . . | 9 |
| Position undetermined in . . . . | 38 |

The local changes in the mark itself are as follows:

| | |
|---|---|
| Accompanied by ecchymosis and subcutaneous extravasation, in . | 21 |
| Of a yellow colour, without ecchymosis or extravasation . . | 50 |
| Unknown . . . . . . . . . . . . . . | 35 |

Thus we learn that, in seventy-one well-ascertained cases of hanging during life, only twenty-one were accompanied by true ecchymosis in the depression produced by the cord, which is in the ratio of about *two cases to seven.* In three of these cases, there was no greater change of colour in the skin, externally, than had been remarked in experiments on the dead. The white or colourless depressions were mostly met with in very fat subjects.

This fact is most important in Medical Jurisprudence: it shows that a ligature in destroying life by hanging, does not necessarily cause a bloody impression; on the contrary, in many cases the mark is precisely similar to that which is produced in the suspension of subjects *soon after death.* The time which the ligature remained around the neck of the individual hanged was observed to make no difference as to the production or non-production of ecchymosis by it. When it was removed sooner or later after death, ecchymosis was sometimes found, and at other times not.

Remer imagined that the absence of ecchymosis in the course of the ligature indicated death by apoplexy; but Casper found that the mark was not ecchymosed, as well when the vessels of the brain were empty as when they were congested. It is generally assumed that, in hanging, death takes place from apoplexy, from suffocation, or from the two kinds of death simultaneously: but this opinion requires some modification. In the 106 cases recorded by Casper, death took place:

| | |
|---|---|
| From apoplexy in . . . | 9 |
| suffocation . . . | 14 |
| both conditions . . . | 62 |
| neither . . . . | 5 |
| Unknown or unexamined bodies . | 16 |

In not one instance of the apoplectic cases was blood effused on the brain: there was merely more or less congestion in the sinuses and vessels. It is in this sense that we are to understand the meaning of the word apoplexy, as applied to death by hanging. The word, however, thus employed conveys an ambiguous meaning. We ought rather to say, that death is caused by an obstruction to the circulation in the brain. The lividity of the countenance depends upon this obstruction; for when the circulation of blood is impeded in the chest before the cerebral obstruction takes place, the face of the hanged person will not be livid. In some instances Casper found it extremely pale.

The emission of semen, or rather of the *liquor prostaticus,* in the male, has been considered a good sign of hanging during life. In seventy-seven cases, Casper met with this emission in nineteen, about *once in three cases.* Evacuation of fæces was observed only four times in 106 cases. Remer asserted that the female organs of generation were often vascular and congested in death from hanging: but Casper found these appearances but once in twenty-nine cases. The erection of the penis in the male may take place more commonly than is imagined: for, unless the examination be speedily made, the organ will again collapse. Guyon saw fourteen negroes hanged; and, at the moment of suspension, erection took place *in each.* In nine of these cases, traces of this erectile state were perceived *an hour* after death.

The author derives the following conclusions from his observations:

1. Death from hanging is in most cases to be ascribed to an obstruction of the circulation.

2. The *mark* produced by the ligature cannot be relied on as evidence of hanging during life.

3. A ligature applied to the neck a few hours *after death* produces the same local changes as are met with in most of those subjects who have been hanged *during life.*

4. These local changes consist in the skin becoming *brown or yellow in the course of the cord,* as if it had been burnt. There may be also a well marked depression. The skin feels and cuts like leather. In more rare cases, i. e. about 1 : 3½, a true ecchymosed impression is produced by the ligature.

5. The mark produced on the neck of a subject hanged long *after death,* presents none of these characters.

6. The kind of *material* used for the ligature does not affect or modify the local changes produced by it.

7. The exact position of the ligature, in relation to the larynx, has but little influence on these local changes.

Since obtaining the above results from his experiments, the author has met with an opportunity of making a practical application of them in a case which involved a charge of murder.

A girl was delivered secretly of a child in a cellar. The body of this child was found some days afterwards, under suspicious circumstances. The account given by the mother, who was accused of the murder, was that she heard her child cry during delivery, but it soon died. She then covered its body lightly over with straw, and left the place. She returned to the cellar in about an hour; and having twisted a few straws into a band she tied this tightly round the neck of her child, in order, to use her own language, "to prevent it from awaking." On examining the child, it was found to be natural and well formed: and it had evidently respired. Apoplexy was assigned by the examiners as the cause of death, and they affirmed that this had most probably resulted from the child having been strangled by the band of straw found around its neck. The accused was condemned to be publicly whipped and to be confined for life.

An appeal was made from this sentence, and our author's opinion was called for. The medical witnesses had stated, in their evidence relative to the mark produced by the band, "that the depressions caused by each straw were clearly seen on the neck. These depressions were *whiter* than the rest of the surrounding skin; but the little folds or elevations of skin between the straws were *red.* On cutting into these red spots, it was found that in some of them the discoloration was owing to true ecchymosis in the cutis." The ecchymosis is stated to have been very slight: but still, from the appearance, the witnesses believed that the mark had been produced during life: in other words, that the child had been born alive and strangled by the mother.

Our author, after observing that no coagulated blood was found in the skin beneath the mark, and then neutralizing the effect of this observation by admitting that such an extravasation is seldom or ever met with in death from strangulation, proceeds to contend that the slight degree of ecchymosis, in the course of the mark in this case, *might* undoubtedly have resulted from the application of the straw-band *soon after death,* and while the body was yet *warm.* From the depositions, it appeared that the child could not have been dead more than *an hour* when the ligature was applied: for the prisoner confessed that she returned and put it round her child's neck, about an hour after her delivery. She might not have been absent so long; but allowing that her statement as to the time was correct, the mark might have been produced, since the body would not during that time have become cold. Hence Dr. Casper declared, that it could not be positively assumed that the child was living when the ligature was placed around its neck: therefore it was not established that it had died by strangulation. In consequence of this declaration, a mitigation of punishment followed.

[Remarks. Among the experiments performed by the author of this paper, the earliest period after death at which a ligature was applied around the neck was an hour: and, in this case, there was no well defined ecchymosis produced by it. In those experiments, in which six, thirteen, and twenty-four hours had elapsed after death before the ligature was employed, the skin remained wholly unaffected. In others, where the individuals had been dead not more than one and two hours, the mark left by the cord presented those characters which are most generally met with in hanging during life: i. e. the skin which had sustained the compression was *hard* and of a *yellow colour*. It still remains to be determined, whether these appearances can be produced at intervals of three, four, or five hours after death, so that the period at which such effects would cease to be produced has yet to be ascertained. It is to be regretted that our author has not stated, whether all of his cases were cases of suicide or not. The mark produced by a cord is generally much stronger, and presents a more decidedly ecchymosed character, in individuals who have undergone the sentence of the law or who have fallen victims to homicidal violence, than in those who have died by their own hands.

Dr. Casper seems to us to have represented the cause of death in its true light. It is clear that if a mere congestion of the cerebral vessels be taken as conclusive evidence of death from apoplexy, two thirds of the cases of death from organic disease, as of the liver, kidneys, or other remote parts of the system, might be with equal justice referred to the same cause. No language can be more vague than that which ascribes death to an apoplectic attack, simply because the vessels and sinuses of the brain are found somewhat full. Our author truly observes that, in hanging, this fulness furnishes evidence of the cerebral circulation having been, from some cause, obstructed: but such an obstruction, where it has existed, is not to be confounded with that morbid condition of the brain which is generally met with in cases of apoplexy.

We agree in all the conclusions of Dr. Casper except the sixth. Whether a cord or cravat be the material of suspension in suicidal hanging, the mark on the neck is generally destitute of ecchymosis, presenting the yellow horny character already described; but we firmly believe from actual observation, and we see nothing contradictory to our views in the cases quoted in this paper, that a hard ligature like a cord is more liable to cause ecchymosis and excoriation, as well as to leave a more visible depression on the neck, than a soft material like a cravat.

We have one remark to make on the case with which Dr. Casper concludes his essay. We learn from the report of this case, that the ecchymosis produced by the ligature on the neck of the child was slight; and that it was only perceptible in the little elevated portions of skin corresponding to spaces between the folds of straw. Now this description of the appearances will apply to a mark produced during life, or, as allowed by Dr. Casper in this case, within *an hour* after death. We admit this latter position from facts with which we are acquainted, relative to the production of ecchymosis in the dead; but we do not find among the experiments performed by the author of this paper, any one which bears out the somewhat exclusive view which he took of the circumstances. In the two experiments where the subjects were hanged within an hour after death, there was not even the semblance of ecchymosis, while in the case of the child there was evident, although interrupted, subcutaneous effusion. We will, however, allow that the ligature might have been put round the neck of this child either living or dead. But which was the most probable circumstance, and whether it were more likely, that a strong ligature should have been thus tightly applied *after death* to prevent a child from "awaking," or *before death* for the assumed purpose of destroying it, were facts upon which, of course, it was for a jury and not for a medical witness to decide. When medical evidence is thus nicely balanced, circumstances alone can establish the guilt or clear up the innocence of an accused party. If there was nothing more in this woman's favour than her own statement, relative to the time at which she applied the straw ligature, and to the object which she had in applying it, we must declare our opinion that, in her appeal, she not only met with a very humane medical witness, but with a very merciful tribunal.]

*Wochenschrift für die gesammte Heilkunde. Januar,* 1837.

